# Different patterns of neurogenic quadrilateral space syndrome: a case series of undefined posterior shoulder pain

**DOI:** 10.1186/s10195-024-00813-y

**Published:** 2025-01-02

**Authors:** Giuseppe Porcellini, Alberto Brigo, Michele Novi, Elisa De Santis, Silvia Di Giacomo, Andrea Giorgini, Gian Mario Micheloni, Rocco Bonfatti, Alessandro Donà, Luigi Tarallo

**Affiliations:** 1 University of Modena and Reggio Emilia, Modena, Italy; 2 Shoulder Team Forlì, Forlì, Italy; 3 Ospedale San Pietro Igneo Fucecchio, Firenze, Italy

**Keywords:** Quadrilateral space syndrome, Axillary nerve, Nerve entrapment syndrome, Posterior shoulder pain, Anterior deltoid atrophy, Overhead pain, Sports injury

## Abstract

**Background:**

Quadrilateral space syndrome is a painful disorder of the shoulder caused by static or dynamic entrapment of the axillary nerve and the posterior humeral circumflex artery. It was first described in 1983; however, it is an uncommon syndrome that initially presents with nonspecific shoulder pain or selective deltoid atrophy, and diagnosis is often delayed owing to its rarity. Young athletes of overhead sports are more commonly affected by this syndrome. Symptoms of quadrilateral space syndrome include silent deltoid atrophy, persistent posterior shoulder pain, paresthesias, and tenderness over the quadrilateral space. Vascular symptoms may involve thrombosis and embolisms of the upper limb. Instrumental tests and imaging are not always conclusive, leading to frequent misdiagnosis of the syndrome.

**Patients and methods:**

The aim of this study is to present a case series of four patients diagnosed with neurogenic quadrilateral space syndrome, describe different clinical presentations, and suggest tips for diagnosing this syndrome. All patients underwent a detailed medical history collection, were interviewed about the sports and hobbies they engaged in, and received a comprehensive clinical examination of the neck and shoulder. Patients also underwent diagnostic exams such as magnetic resonance imaging (MRI) and electromyography. An ultrasound-guided injection of local anesthetic was performed into the quadrilateral space.

**Results:**

All patients affected by neurogenic quadrilateral space syndrome underwent conservative treatment, which included a rehabilitation program. Only one out of four patients experienced complete resolution of symptoms and did not require surgical decompression.

**Conclusions:**

To properly treat this rare syndrome, we propose classifying it as either “dynamic” or “static,” on the basis of the clinical history, MRI findings, and physical examination. The study includes a rehabilitation program that was effective for one patient, demonstrating that surgical decompression may be avoidable if the cases are promptly diagnosed and classified.

*Level of evidence* IV according to “The Oxford 2011 Levels of Evidence”

## Introduction

Cahill and Palmer first described the entrapment syndrome of the axillary nerve (AN) and the posterior humeral circumflex artery (PHCA) within the quadrilateral space (QS) in 1983 [1]. The QS is bounded by the upper border of teres major inferiorly, the lower border of teres minor superiorly, the long head of triceps medially, and the surgical neck of the humerus laterally [1]. Within the QS, the axillary nerve divides into three distinct fascicular groups that innervate different fibers of the deltoid and teres minor. Several anatomic variants have been described [2–5]. Notably, in approximately 14% of individuals, the long head of triceps brachii is partially or entirely innervated by a branch of the axillary nerve [6, 7].

It predominantly affects young male athletes (20–40 years old) who perform shoulder abduction and external rotation (AER) movements, such as swimmers and tennis, volleyball, and baseball players [8]. Athletes are at higher risk for dynamic stress on the axillary nerve compared with the general population; the presumed prevalence in a cohort of beach volleyball athletes was estimated to be 2.2% [9, 10]. Fibrous bands or other space-occupying lesions can be responsible for the symptoms [10].

Quadrilateral space syndrome (QSS) can present with neurological signs and symptoms such as posterior shoulder pain, nondermatomal paresthesias, tenderness over the quadrilateral space, and isolated deltoid atrophy [11]. The epidemiology of QSS is not fully understood; a small number of cases have been described so far, and the heterogeneity of clinical presentation makes the diagnosis challenging even for senior clinicians, as posterior shoulder pain is frequently vague and difficult to interpret.

It is worth noting that all the patients described in our case series were competitive athletes in overhead sports. This consideration helped us in making the diagnosis and choosing the appropriate treatment. Moreover, when QSS affects the posterior humeral circumflex artery, known as “Pitcher syndrome,” symptoms may include thrombosis, aneurysms, embolisms, and digital or hand ischemia [8, 10]

### Pathogenesis, clinical features, and diagnosis

The pathogenesis of this syndrome is multifactorial; anatomic findings in neurologic QSS include paralabral cysts, fibrous bands, venous plexus, and space-occupying lesions such as osteochondroma, lipomas, and axillary schwannomas [8]. In vascular QSS, repetitive AER movements induce turbulent blood flow, leading to thrombotic occlusions and aneurysms. In neurogenic QSS (nQSS), the undefined shoulder pain may be aggravated by AER movements [8]

Some cases of painless QSS have also been described in beach volleyball athletes that presented anterior deltoid atrophy and weakness in forward flexion. It is frequently associated with atrophy of the teres minor, though cases with normal or hypertrophic teres minor are also observed [11].

A complete clinical assessment of all common causes of posterior shoulder pain is crucial for making a diagnosis. We suggest including the evaluation of posterior labral injury, posterior instability, and suprascapular nerve entrapment.

The Mayo Clinic classifies QSS into neurogenic and vascular types [10]. Neurogenic QSS includes silent deltoid atrophy, persistent shoulder pain, nondermatomal paresthesias, tenderness over the quadrilateral space, movement limitations unrelieved by common painkillers, and fasciculation of the posterior deltoid or long head of the triceps brachii.

Vascular quadrilateral space syndrome encompasses thrombosis and embolisms, characterized by pallor, coolness, and cyanosis of the hand and digits.

Tenderness over the quadrilateral space suggests QSS, confirmed by ultrasound-guided injection of 5 mL of 1% plain lidocaine into the quadrilateral space. If the anesthetic spreads between the infraspinatus and the teres minor, the test might not be specific, as the anesthetic could involve both the suprascapular nerve and the axillary nerve [12]. To avoid misleading examinations, the procedure should be performed by an expert ultrasound technician. Electromyography (EMG) is not always indicative of QSS because the condition is sometimes caused by a dynamic compression within the quadrilateral space muscular belly, thus the examination should be performed during AER movements. When EMG is performed at rest, it can exclude suprascapular nerve entrapment or significant axillary nerve involvement [13]. MRI is useful for evaluating cystic ganglion or space occupying lesions, and examining the muscle belly may show the first signs of denervation or nerve damage, such as muscle edema or atrophy of the teres minor and deltoid muscle. Two weeks after denervation, it is possible to observe diffused belly edema on STIR images, which sometimes are the first signs of nerve distress [14]

## Patients and methods

We present a series of four clinical cases that were consecutively diagnosed and treated as neurogenic quadrilateral space syndrome (nQSS) in the last 5 years; none of the patients exhibited features of vascular QSS, which in our experience, is a very rare condition. All patients underwent a detailed medical history collection, were interviewed about the sports and hobbies they engaged in, and received a comprehensive clinical examination of the neck and shoulder, including static and dynamic tests for disc herniation, rotator cuff tendinopathy, glenohumeral instability, capsulolabral tears, scapular dyskinesis, and shoulder stiffness. All patients also underwent magnetic resonance imaging (MRI) and electromyography (EMG). To complete the diagnostic process, an ultrasound-guided injection of local anesthetic was performed into the quadrilateral space by clinicians well trained in shoulder ultrasound examination. After confirming quadrilateral space syndrome, all patients underwent conservative therapy, which included a rehabilitation program and the administration of painkillers.

The conservative treatment focused on strengthening the scapular stabilizing muscles, along with the posterior deltoid and restoring shoulder rotator balance, and to prevent scapular protraction and improper mobility. Reinforcement of pectoralis major and subscapularis muscles was used to compensate for the lack of anterior deltoid function. Transverse friction massages were also used to release soft tissue stiffness.

Finally, self-assisted stretching exercises for the posterior capsule were proposed.

**Fig. 1:**
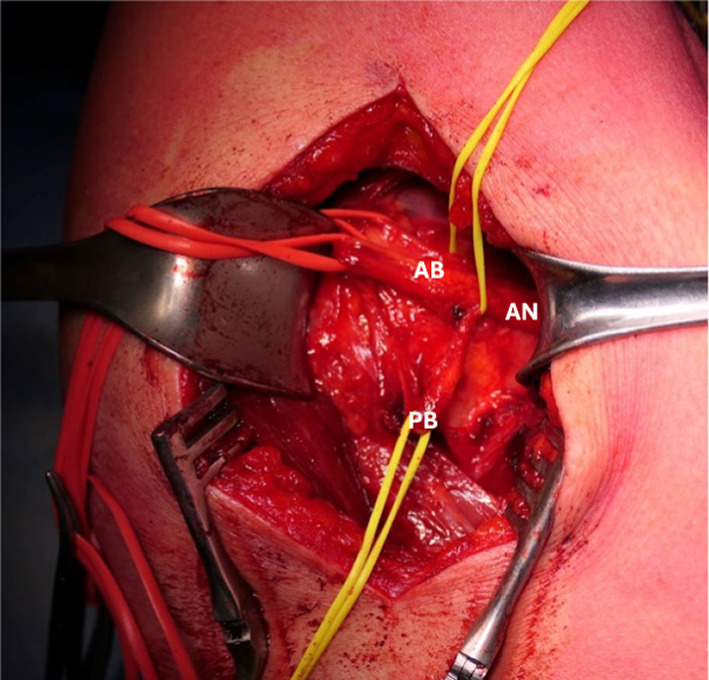
Surgical approach to the QS; it is possible to identify the common branch of axillary nerve (AN), which divides into the branch for anterior and middle deltoid (AB) and in the posterior branch (PB) for posterior deltoid and teres minor. In this picture, it is possible to observe the hourglass deformation of the PB, which was entrapped by fibrous bands

If surgical procedure is considered necessary, we suggest the lateral decubitus position: a 5-cm surgical incision is made, centered on the posterior border of the deltoid. Using retractors to gently elevate the deltoid, the adipose tissue within the quadrilateral space is identified, and by blunt dissection, the axillary nerve is isolated in its various components as described in Fig. 1.

The intraoperative examination with an electroneuro stimulator allows us to identify the various branches of the axillary nerve and to assess the potential for partial or complete paralysis of these branches.

**Fig. 2:**
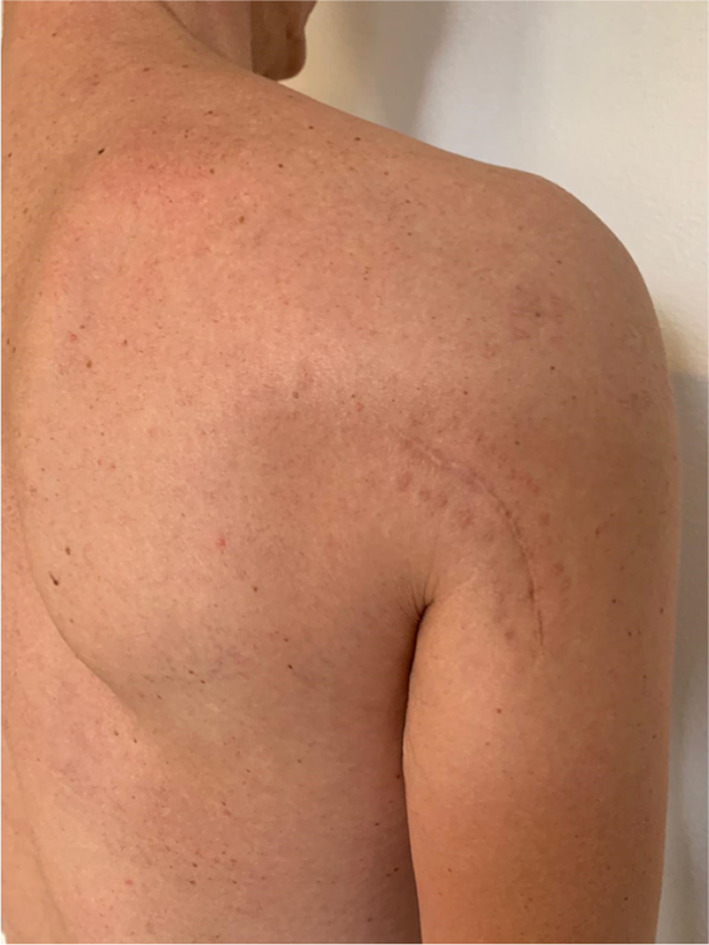
Surgical approach to the quadrilateral space

**Fig. 3:**
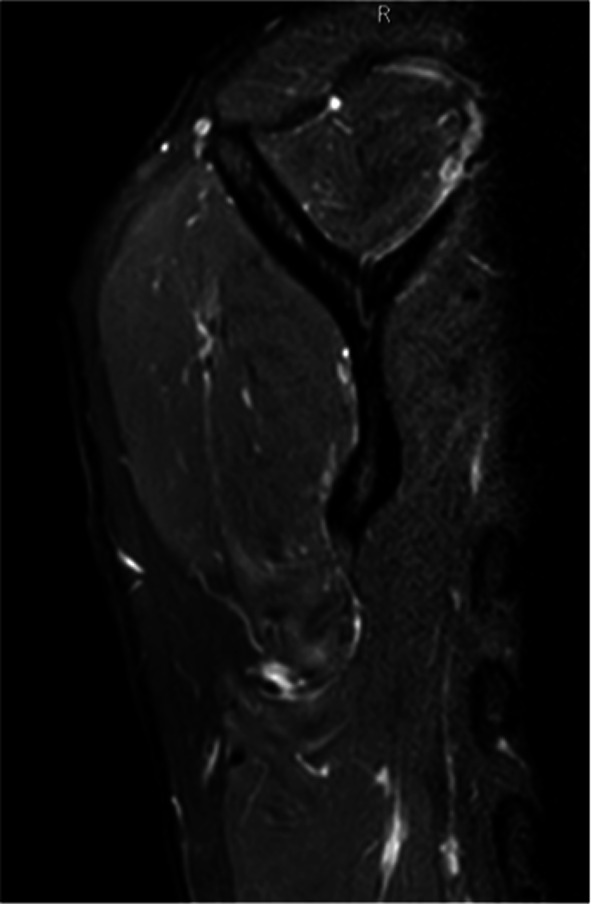
Edematous and atrophic right teres minor on magnetic resonance imaging of shoulder

**Fig. 4:**
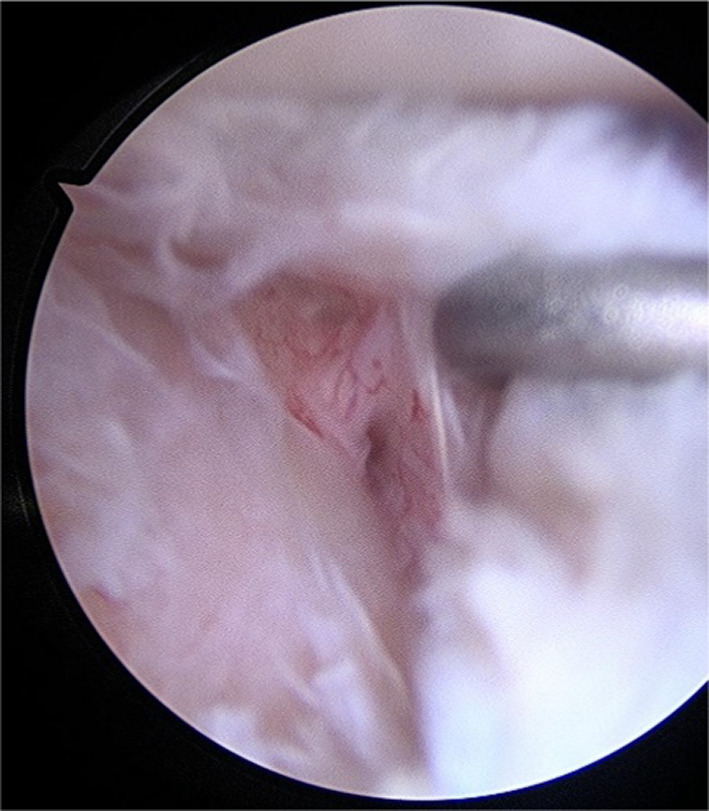
Posterior labral peel-back lesion that is elevated by a palpator tool

**Fig. 5:**
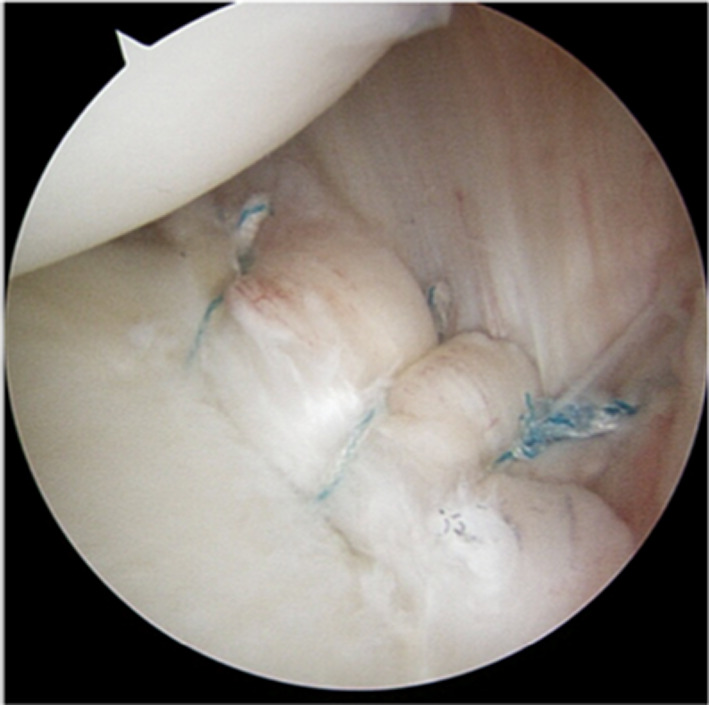
Anchors repair of posterior labral peel-back lesion

The rehabilitation program after surgical decompression of quadrilateral space was tailored to each patient; initially, activation of periscapular and rotator cuff muscles was encouraged to restore shoulder balance. When possible, early mobilization was recommended to limit the formation of fibrous bands. Patients who underwent surgical neurolysis were evaluated the day after surgery, as well as 1 month and 3 months postoperatively [13]. Patients receiving conservative treatment underwent strict clinical evaluation to monitor their progress. Follow-up concluded once the patient reported resolution of symptoms (visual analog score [VAS] 0–1) and complete recovery of active and passive range of motion (ROM), measured in comparison with the contralateral shoulder (Figs. 2, 3, 4, 5).

### Case presentation

#### Case 1

A 54-year-old right-handed male tennis player presented with posterior shoulder pain, paresthesias, and limitations in daily activity, sports practice, and sleeping. The visual analog scale (VAS) was 6 at rest and 9 during AER movements. Symptoms worsened insidiously, and the patient used a sling for pain management. Clinical examination ruled out rotator cuff disease and cervical pain but revealed a posterior clicking and a positive Porcellini’s test [15]. The pain was intensified by palpation over the quadrilateral space and pain relief after ultrasound-guided lidocaine injection (lido-test) confirmed QSS. No differences in deltoid muscle trophism were noted. He underwent electromyography, which did not detect abnormalities of the axillary or suprascapular nerves. The administration of oral nonsteroidal antiinflammatory drugs, opioids, and even pregabalin was not completely effective. The pain intensified during rehabilitation exercises for the deltoid and teres minor, and therapeutic massages did not improve the symptoms. After some sessions, the pain worsened, and physical therapy was interrupted. MRI showed no abnormalities; 2 months later, he repeated the examination, and a progressive atrophy of the teres minor was noted in the comparative view of the sagittal plane.

Four months after the onset of symptoms and relative conservative therapy, the patient underwent surgical decompression of the fibrous band entrapping the branch for the teres minor. An arthroscopic view was also performed, and posterior labral peel-back lesion was fixed with anchors. The patient reported complete pain relief the day after the surgery and began a rehabilitation program, which involved activating the periscapular muscles and resting for 3 weeks in a sling with 15° abduction pillow. After 3 weeks, passive shoulder mobilization was initiated across all planes, with internal rotation avoided for an additional 3 weeks. Following the restoration of range of motion, the patient was encouraged to begin active movements in a warm pool. Eight weeks post surgery, the patient was ready to start a strengthening program for the external rotators, deltoid, and humeral head depressor muscles. Subsequently, the internal rotator muscles were also strengthened to prevent imbalance. Only after isometric testing confirmed balanced strength between internal and external rotators did the patient return to play.

#### Case 2

A 17-year-old right-handed professional volleyball player presented with undefined shoulder pain and loss of strength and control during shoulder forward elevation in hitting and service actions. During the clinical examination, atrophy was observed in the anterior deltoid, along with a significant reduction in shoulder flexion strength, rated as grade 4 on the Medical Research Council (MRC) scale. Range of motion (ROM) was preserved but the subscapularis was 40% weaker than the contralateral side. Dynamometric measurement showed a significant imbalance between internal and external rotations, favoring the external rotators. An ultrasound-guided Lido-test into the QS was positive.

**Fig. 6:**
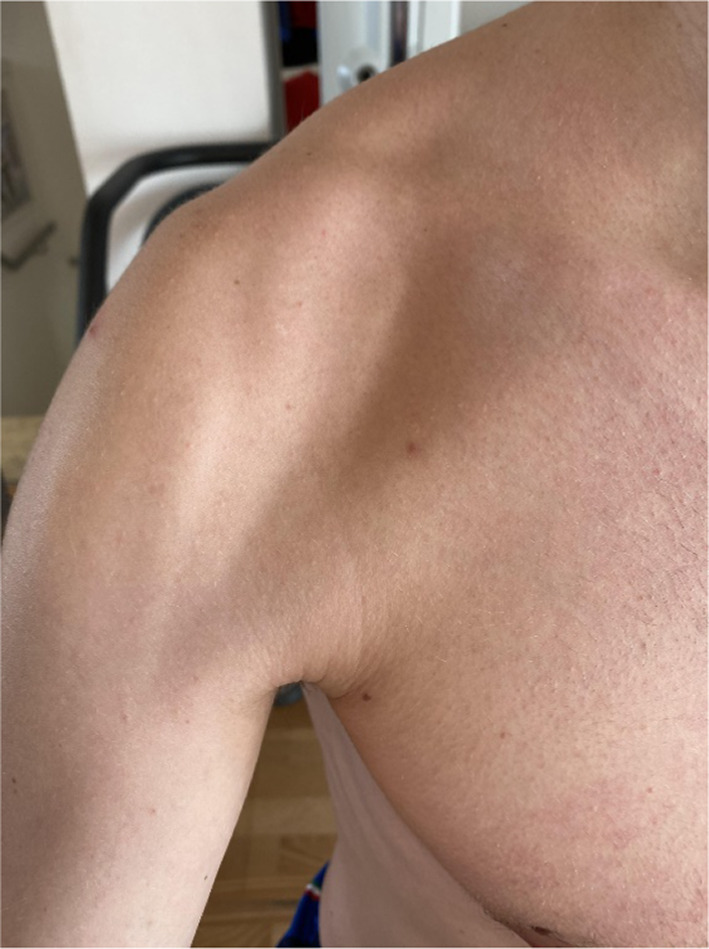
Anterior deltoid atrophy in a young volleyball player

**Fig. 7:**
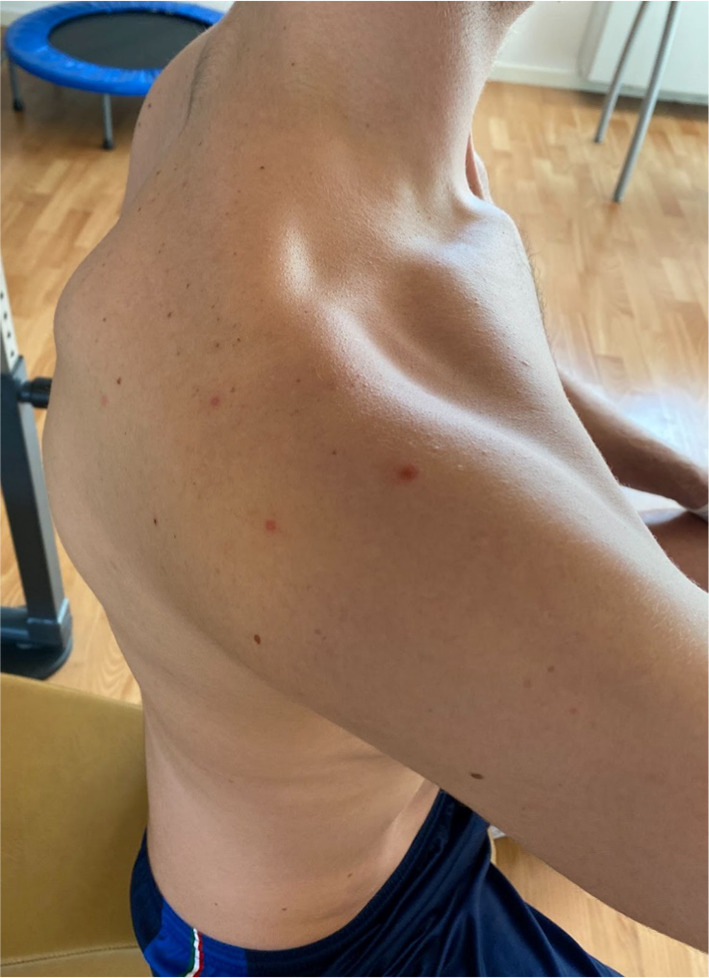
Anterior deltoid atrophy in a young volleyball player

**Fig. 8:**
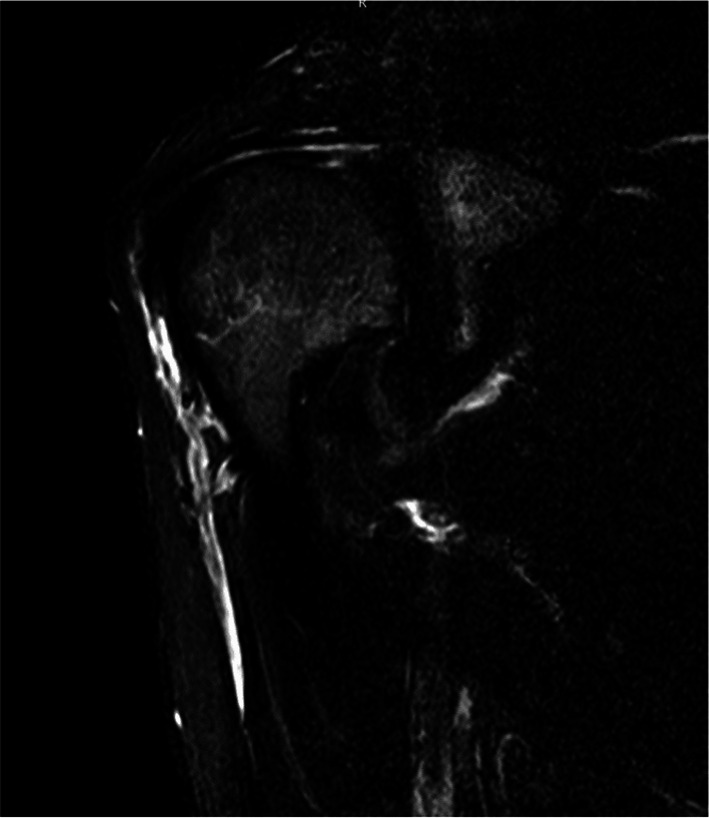
Anterior deltoid atrophy shown by magnetic resonance imaging

MRI highlighted anterior deltoid atrophy, whereas EMG revealed inactivity of the axillary nerve. The patient stopped volleyball practice for eight weeks and began strengthening the scapular stabilizers, rotator cuff muscle, and restoring shoulder rotator balance [16]. Reinforcement of pectoralis major and subscapularis muscles was used to compensate for the lack of anterior deltoid function. After 6 months of physical therapy, the patient returned to previous volleyball performance levels. In the first year of follow-up, the patient continued to play as a professional spiker in the highest tier of the Italian volleyball league. (Figs. 6, 7, 8)

#### Case 3

A 22-year-old right-handed female volleyball player presented with nontraumatic shoulder discomfort during sport activities, weakness and limitation in active forward flexion, and abduction without pain . Anterior deltoid atrophy had been evident for the previous 3 months, and rotator cuff tests were negative. Both the palpation of quadrilateral space and the lido-test were positive.

The electromyographic (EMG) examination revealed spontaneous fibrillation potentials in the anterior part of the deltoid and partial involvement of the teres minor. A hyperintense area was noted around the axillary nerve within the quadrilateral space, but no further signs of rotator cuff disease were observed by MRI examination.

She underwent 6 months of conservative treatment, including physical therapy, nonsteroidal antiinflammatory drugs (NSAIDs) and acetyl-l-carnitine, without resolution of symptoms. The anterior deltoid atrophy worsened during that period.

Surgical decompression of the axillary nerve in the QS was performed by releasing the fibrous bands from the long head of the triceps to the teres major. From day 1 after surgery, strength against resistance during isometric tests for teres minor and deltoid muscle improved significantly and 1 month later was measured as 5 points out of 5 on the MRC scale. During follow-up, the deltoid atrophy did not improve; however, range of motion was complete and symmetrical to the opposite side, and the patient did not report any limitations in daily living or sport activities. She returned to playing after 4 weeks of rehabilitation.

#### Case 4

A 36-year-old volleyball player and amateur cyclist was treated for a severe adhesive capsulitis and recovered in about 2 months. Six months after the resolution of symptoms, progressive atrophy of the anterior deltoid was observed. The deltoid strength was evaluated 4 points out of 5 on the MRC scale. The patient presented with pain, weakness, complete passive ROM but limitation in active ROM, and negative rotator cuff tests. Hypoesthesia of the axillary nerve area was reported by the patient. The EMG examination revealed a reduction in compound muscle action potentials (CMAP), suggesting chronic neurogenic damage without signs of active denervation. The MRI did not show any rotator cuff disorders except for edema of the teres minor and adipose degeneration of the muscle belly.

He was initially treated with a physical therapy program, NSAIDs, prednisone, and acetyl-l-carnitine. After 6 months of conservative treatment, there was a worsening of pain, weakness, and anterior deltoid atrophy. Surgical decompression of the axillary nerve in the quadrilateral space was performed by removing a large fibrous band tense between the long head of the triceps to the teres major. From the first day after surgery, the patient experienced surprising immediate relief from shoulder pain and recovery of the deltoid strength (5/5 MRC scale) during isometric tests against resistance. These improvements were confirmed and continued to improve after rehabilitation and 1 month of follow-up.

## Results

This case series presents four cases of neurogenic quadrilateral space syndrome (QSS). The oldest patient was 54 years old, and all patients were competitive athletes in overhead sports involving abduction and external rotation movements. The group included three males and one female, with one case being a professional volleyball player, who was the only patient successfully treated with conservative management in the series. The outcomes considered were pain relief and functional recovery of the shoulder movements; all the patients returned to prior strength and performance levels and complete active and passive range of motion after different rehabilitation programs.

## Discussion

QSS is considered rare, and prevalence is underestimated owing to the complexity of its diagnosis and to the considerable percentage of asymptomatic patients [11].

Frequently, clinicians arrive at a QSS diagnosis after excluding more common diseases, sometimes considering QSS only after unsuccessful surgery. If properly diagnosed, surgery is not always necessary for this disease.

In our experience, young athletes involved in overhead activities that present a posterior shoulder pain are eligible for QSS differential diagnosis.

We present four clinical cases of neurogenic QSS and describe different shades and aspects of these patients.

In cases 1, 3, and 4, the fibrous bands were so tenacious that the conservative therapy did not provide pain relief. In case 1 and 4, the exercises worsened the symptoms, necessitating interruption. The triggering factors in case 1 and 4 were posterior labral peel-back lesion and adhesive capsulitis, respectively. Case 3 might have benefited from conservative therapy, similarly to case 2, if initiated before the onset of severe symptoms. Case 2, likely caused by dynamic compression of the axillary nerve between the deltoid and hypertrophic teres minor, responded well to rest and rehabilitation without further treatment.

Volleyball players frequently experience shoulder issues, and dynamic QSS shares aspects with the “hitting shoulder,” caused by traction on suprascapular nerves. Both conditions lead to selective muscle atrophy, neurological pain, and functional limitation [17]. Prompt physical therapy is crucial, and surgery is not always indicated, with the timing of intervention being debatable.

Before planning a surgical decompression of the axillary nerve, a minimum of 6 months of rest and conservative treatment, including NSAIDs, physical therapy for therapeutic massage, and exercises to strengthen scapular stabilizers and stretch of the posterior shoulder capsule, is recommended. Strengthening periscapular muscles restores shoulder balance and prevents further trauma to the QS entities [8, 13, 18].

Studies on the efficacy of physical therapy techniques are lacking, and rehabilitation programs need to be personalized according to the patient’s symptoms and needs. Sometimes the intensity of pain is so important that physical therapy cannot be carried out. When pain is under control, it is important to maintain muscle trophism of the anterior deltoid and teres minor with recruitment exercises and electrostimulation [19, 20]. In case 2, the atrophy of anterior deltoid was balanced with the training of pectoralis major and subscapularis muscle to strengthen the anterior wall of the shoulder; the treatment was effective and the patient returned permanently to high performance.

Cases 3 and 4 had a marked improvement in strength during postoperative isometric tests of both teres minor and deltoid; in case 3, the strength was symmetrical the day after the surgical procedure.

According to the most recent literature [8] and our clinical experience, we propose a classification for cases of QSS that have a neurological impairment:

- 1. “Static” QSS: this type is caused by fixed structural entities exerting external compression on the quadrilateral space. Cahill and Palmer identified fibrous bands as the primary cause, which form as adhesions due to repeated microtrauma to the quadrilateral space. These bands are often observed between the long head of the triceps and the teres major in cadaveric studies. Other space-occupying lesions, such as paralabral cysts, lipomas, humeral osteochondroma, axillary schwannomas, and venous plexus can also contribute to the syndrome.
- 2. “Dynamic” QSS: this type is associated with muscle hypertrophy around the quadrilateral space, particularly in competitive athletes engaged in overhead sports. Repetitive movements of abduction and external rotation can lead to dynamic entrapment of the axillary nerve.

Limitations of this study are the low number of cases and the multiple factors that may be underestimated when treating a rare syndrome.

**Fig. 9:**
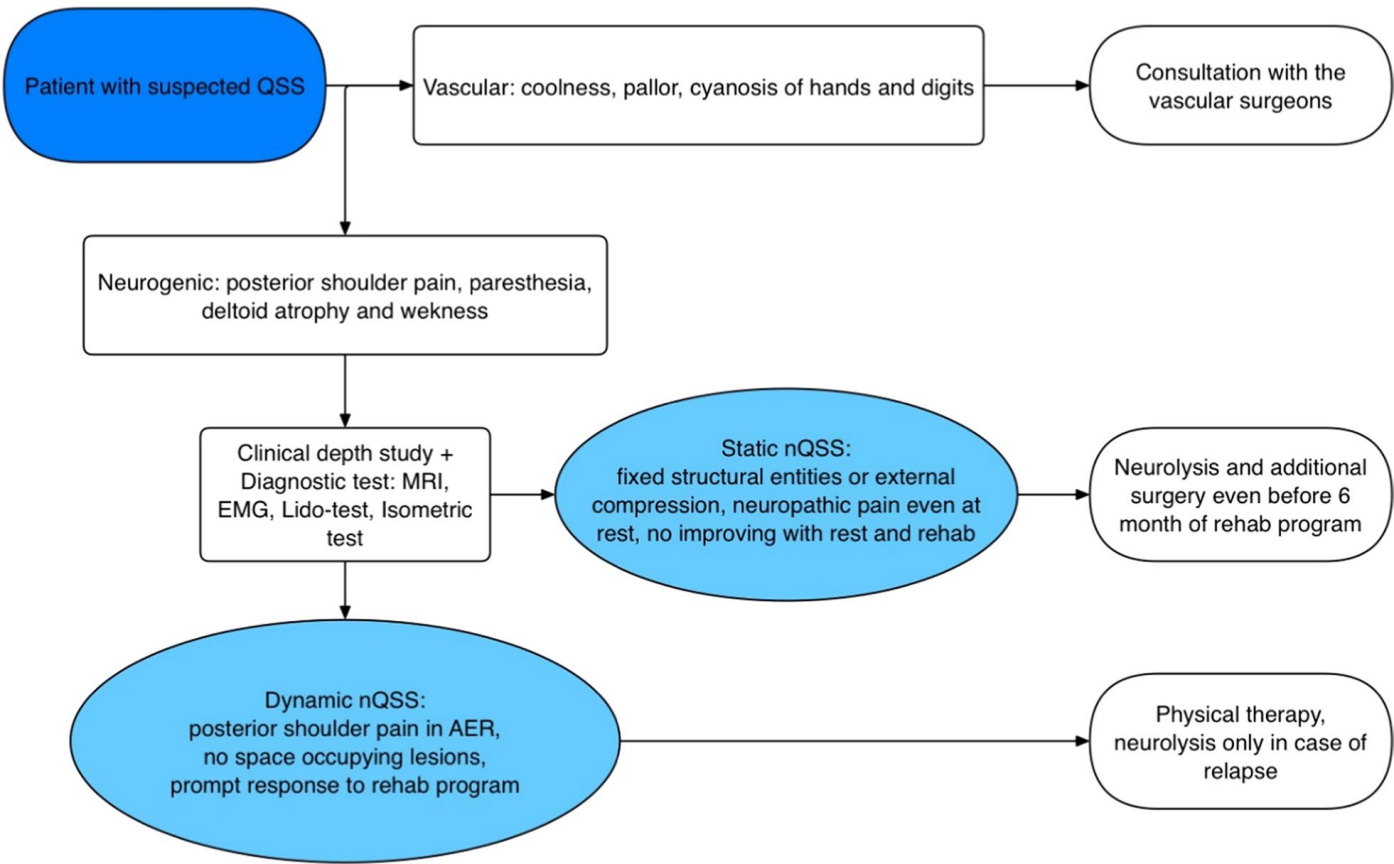
Diagnosis and treatment flowchart for patients with suspected QSS. *QSS* quadrilateral space syndrome, *MRI* magnetic resonance imaging, *EMG* electromyography, *Lido-test* lidocaine block of the axillary nerve into the QS, *nQSS* neurogenic QSS, *vQSS* vascular QSS, *AER* abduction and external rotation

In case 1, a limitation is that two different surgical procedures were performed during the same surgery, and we could not assess whether the pain relief derived from axillary nerve decompression or from the posterior labral lesion. However, we are confident to assess that the neuropathic paralyzing pain that was described by the patient before the surgery was not consistent with a problem of glenoid labral disruption. (Fig. 9)

## Conclusions

Quadrilateral space syndrome (QSS) is a little-known condition in terms of diagnosis and treatment. The Mayo Clinic proposed an innovative classification of QSS based on the prevalence of neurogenic or vascular symptoms. We hope that clinicians may find the new subclassification we presented helpful for diagnosing specific types of QSS and providing appropriate treatment.

This classification suggests that isometric tests alone may no longer be sufficient for the clinical evaluation of unexplained posterior shoulder pain. The biomechanical analysis of athletic movements, along with wide clinical evaluation using dynamic tests, is becoming increasingly important. The ultrasound-guided lidocaine test into the quadrilateral space is the examination that best completes the clinical evaluation; however, it should be entrusted to experts.

There is still much to learn, and further studies are needed to determine the optimal timing for surgery. We question the established 6-month period of conservative treatment before considering surgery: if signs and symptoms are severe and the suspicion of quadrilateral space syndrome is well founded, surgical intervention should not be delayed.

Additionally, criteria for defining full recovery, particularly for overhead athletes, remains to be established. While clear guidelines are still lacking, we recommend dynamometric evaluation of bilateral internal and external rotators, to strengthen the deficient rotators and restore shoulder balance before returning to play.

## Data Availability

Not applicable.

## Abbreviations

QSS: Quadrilateral space syndrome
QS: Quadrilateral space
nQSS: Neurogenic quadrilateral space syndrome
ROM: Range of motion
AN: Axillary nerve
PHCA: Posterior humeral circumflex artery
AER: Abduction and external rotation
EMG: Electromyography
STIR: Short tau inversion recovery Medical Research Council scale
VAS: Visual analog scale
NSAIDs: Nonsteroidal antiinflammatory drugs
CMAP: Compound muscle action potentials
MRC: Medical Research Council scale
